# Pattern of acute organophosphorus poisoning at University of Gondar Teaching Hospital, Northwest Ethiopia

**DOI:** 10.1186/s13104-017-2464-5

**Published:** 2017-04-04

**Authors:** Getnet Mequanint Adinew, Assefa Belay Asrie, Eshetie Melese Birru

**Affiliations:** Department of Pharmacology, School of Pharmacy, College of Medicine and Health Science, University of Gondar, P. O. Box: 196, Gondar, Ethiopia

**Keywords:** Antidote, Atropine, Management, Organophosphate, Poisoning

## Abstract

**Background:**

Despite the apparent benefits of organophosphate compounds (OPCs) acute organophosphate (OP) pesticide poison is an increasing problem worldwide. In a country like Ethiopia, where agriculture is a major component of the economy, these compounds are readily available to the general public. There is paucity of evidence from Ethiopia showing the pattern of organophosphate poisoning (OPP) in healthcare facilities. The objective of this study was to evaluate retrospectively the pattern of acute OPP at the University of Gondar Teaching Hospital in northwest Ethiopia, during September 2010 through December 2014 was conducted. Data was collected through chart review of patients who were admitted due to poisoning. Data was analysed using SPSS 20.

**Results:**

Organophosphate poisoning in University of Gondar teaching hospital accounts for about 38.46% of all emergency room admissions for poisoning. Out of the 90 cases studied 60% (54) were women, with male to female ratio of 1:1.5. The mean age of the patients was 25.5 with a standard deviation of 9.45. 56.7% of the cases studies lived in an urban environment compared to 43.3% who lived rurally. In the vast majority of patients, 90% (81) patients had ingested OP as an act of suicide. Regarding the route of exposure, oral ingestion was most common in suicidal cases (88.9%). The elapsed time between the time of poison ingestion and the start of the treatment, ranged from 13 min to 1 day. Health care professionals’ useds decontamination methods such as gastric lavage and activated charcoal (45.6%) and 36.7% use atropine for OPP treatment. The mean hospital stay was 0.74 days. In the present study family problems were a leading cause of suicides and accounted for 45.8% of all cases.

**Conclusion:**

As a developing nation who economy relies heavily on agriculture, Ethiopia continues to have OP compounds remain a common cause of acute poisonings. This is particularly concerning for younger generation who have high rates of OPP and whose numbers continue to raise. This data suggests that it is essential to strengthen Ethiopians regulatory policy concerning the availability of OPCs. Additionally, it will be important to design an appropriate health education program for the prevention of both suicidal and accidental OPPs for the benefit of the public at large.

**Electronic supplementary material:**

The online version of this article (doi:10.1186/s13104-017-2464-5) contains supplementary material, which is available to authorized users.

## Background

Poisoning is one of the major causes of hospitalization through the emergency department and is a major public health problem. Insecticides are one of the major source of poisonings, of which OPCs are the most common [1]. Even though some of the most important biochemicals are organophosphates, including DNA, RNA and many cofactors that are essential for life, other organophosphates are potent nerve agents, which function by inhibiting the action of acetylcholinesterase (AChE) in nerve cells [2]. The main commercial use of organophosphates is pesticides/insecticides which control or eradicate insects found on food, commercial crops, domestic animals and as infestations in domestic and commercial buildings [3]. The widespread availability of OP-containing household and occupational products provides significant opportunity for intentional and incidental poisonings [4]. Despite the apparent benefits of these uses acute organophosphorus pesticide/insecticide poisoning (OPP/IP) is an increasing worldwide problem [5]. The worldwide number of OPPs is estimated at 3,000,000/year [2]. More than 200,000 of these poisonings occur in developing countries [4]. In Africa, some incidences of OPPs have been reported. Data from Zimbabwe showed that OP self poisoning accounted for around three quarters of hospital admissions for suicidal behaviour and findings from Malawi implicated pesticide ingestion in almost 80% of suicides [6]. In Ethiopia OP pesticides were responsible for the majority of deaths particularly from rural areas [7] and is one of the most common types of poisoning in Ethiopia for suicidal intent [8]. Information regarding organophosphorus compound poisoning (OPCP) in a particular region will help in early diagnosis and treatment of cases, thus decreasing the mortality and morbidity rates. Information available in northwest Ethiopia in regard to OPP is limited. Hence this study was carried out with the objective to find out the pattern of OPP cases at the University of Gondar Teaching Hospital, in Gondar, Ethiopia.

## Methods

This study is a retrospective study of all OPP cases, admitted and managed in the emergency department of University of Gondar Teaching hospital, during September 2010 to December 2014. This hospital serves population of more than 5 million and has a capacity of 400 beds. Data was collected by chart review of patients admitted because of poisoning. The data was analysed using SPSS 20. Documents relating to poisoning cases admitted during the study periods were sorted out and used to fill in a questionnaire. The key information recorded in the questionnaire format included patient gender, age, season of admission, time of ingestion, reasoning of poisoning, type of poison, mode of poisoning, poisoning route, patient status, treatment, duration of hospital stay and outcome of the treatment (the questionnaire format was attached as an Additional file 1). Cases which has incomplete information on patients registration book would be excluded in the study.

## Results

During the study period, 30,154 people were admitted to Emergency department at the University of Gondar teaching hospital. 234 patients were identified to have suffered a poisoning, 90 or 38.46% of these patients were poisoned by Ops. During the study period, the incidence of OPP was 5.6 and OPPs accounted for 0.29% of the total emergency room admissions. The distribution of the cases with regards to sociodemographic characteristics is presented in Table 1. Out of the 90 cases, 60% (54) were women, with a male to female ratio of 1:1.5. The mean age of the patients was 25.8, with a standard deviation of 9.77. The minimum and the maximum age was 14 and 57 years respectively. The majority of these patients were in the age group 21–30 years (42.2%) followed by 11–20 years (38.9%) with progressively smaller percentages with increasing age. 56.7% of the patients lived in urban settings and 43.3% lived rurally. 90% (81) of all the OPP cases were from suicidal acts (Table 1). The status of the patients was 57.8% conscious, 22.2% unconscious and in 20% the status was unknown. The most common forms for ingestion were solid (50.0%), liquid (44.4%), followed by Unknown dosage forms (5.6%). Regarding the route of poisoning, the oral route was the most common in suicidal patients because of the ease of ingestion and the ability to ingest the poison without assistance. In the present study, the oral ingestion was the most preferred (88.9%) overall. Inhalation was usually accidental, rarely suicidal. In the current study, only 2.2% of the total cases are due to inhalational poisoning and 8.9% were unknown. In general, it was difficult to understand where the OP poisons were sourced; the source was unknown in 88.9% of the cases. Of those that were known, the home was most common, accounting 10% and shop (1.1%). This is expected as OPs are often stored in an important manner due to lack of awareness of their hazards. Another 1.1% of the Ops were sourced from shops. The time elapsed between the time of poison ingestion until the start of treatment ranged from 13 min to 1 day. The majority of the cases reached the hospital with 3–12 h (31.1%, Table 1) with a mean time interval of 5.13 h. Out of the 90 patients in the study, only 36.7% patients received atropine, and only 30% received gastric lavage. The common treatment regimens are presented in Table 1. The mean hospital stay was 0.74 day. There was no reported death from poisoning during the study period. The yearly breakdown of all the 90 cases of OPP is 8, 20, 10, 13 and 39 respectively from the year 2010 to 2014. 32.2% of the cases fell in Ethiopia’s spring season between the months of September and November. 26.6% [24] cases fell between the months of September and October; this period is considered the most suitable months for farming. In the present study, family problems were a leading cause of suicides, accounting for 45.8% of cases (Table 2). Accidental ingestion was reported in only two cases, and both had been for the purpose of traditional medicine (the utilized data was attached as an Additional file 2).

**Table 1:** Sociodemographic and clinical characteristics

Age in year	Mean ± SD	Gender	No. cases (%)	Duration of hospital stay		Time elapsed		Domicile	No. cases (%)		No. cases (%)
				Day	No. cases (%)	Hour	No. cases (%)				
11–20	17.97 ± 1.59	Female	54 (60%)	<1	55 (61.1%)	≤2	38 (42.2%)	Urban	51 (56.7%)	Atropine	33 (36.7%)
21–30	25.16 ± 2.77	Male	36 (40%)	1–2	25 (27.8%)	3–12	28 (31.1%)	Rural	39 (43.3%)	Common treatment regimen	
31–40	35.67 ± 3.16	**Mode**		>2	3 (3.3%)	>12	12 (13.3%)			Decontamination (Charcoal and Gastric lavage)	41 (45.6%)
41–50	47.00 ± 2.94	Accidental	2 (2.2%)	Unknown	7 (7.8%)	Unknown	12 (13.3%)			Antacid and simple observation with fluid stabilization	16 (17.7%)
51–60	56.00 ± 1.41	Suicidal	81 (90%)

**Table 2:** Factors responsible for OPP

Factors	Frequency (%)
Family disharmony	38 (45.8%)
Marital disharmony	14 (16.9%)
Unsuccessful love affairs	6 (7.3%)
Domestic violence (pregnant after raped)	4 (4.8%)
For traditional medicine	2 (2.4%)
Mental disorder	7 (8.4)
Being RVI	4 (4.8%)
Conflict in work area	4 (4.8%)
Financial problem	4 (4.8%)
Total	83 (100%)

## Discussion

The use of OPCs is widespread in developing countries like Ethiopia to increase the yield of agriculture products to meet the mounting demand. The increased availability of OPCs has increased the incidence of ingestion, resulting in increasing suicidal and accidental poisoning [2]. OPCs are popular insecticides and pesticides because of their effectiveness and non-persistence in the environment or body owing to their unstable chemical as do organochlorides nature [9]. OPP is an important preventable public health problem in developing countries [10]. However, OPP suicides remain a major clinical problem in developing countries, and are responsible for more than 90% of total poison exposures [1]. Most of the studies from Ethiopia [11] and other countries [12] showed that suicide was the most common reason for the non-accidental poisoning. In this it was 90%. The fact that the majority of cases were due to suicidal mode of poisoning because of their accessibility of OP-products in the market has lead to significant increase for intentional poisoning for getting relived from family quarrels and/or financial burdens [13]. In a study on India by Zaheer et al. it was found that most people believe that poison terminates life with minimal suffering [14]. The analysis of data suggests that the incidence of consumption of OP was 38.46%, which is supported by other researchers’ manuscripts. Nearly half of the admissions to the emergency department with acute poisoning were due to OPCP [15]. The most commonly consumed OP was Malathion (41.1%), which is similar with some other studies [16], the remaining 58.9% was unknown OPCs. However in other studies chlorpyrifos and methyl parathion was found to be the most common [17].

The present study, found that more OPP patients were from the urban areas, when compared to other studies. This can be explained by the relatively easy accessibility of OPCs in urban areas. Additionally, a study conducted in Jimma, Ethiopia like our finding, reported that urban dwellers are more likely to commit suicide than those from the rural areas [11].

The majority of the patients (81.1%), were in the age group 11–30 years, which is similar to that reported by other studies where two-thirds of patients were aged less than 30 years [18]. The distribution of the cases with regard to their age clearly shows that the majority of the cases fall under the productive age group. This age group represents the part of the population which was the most physically, mentally and socially active, which makes them prone to increased levels of stress. This age group often bears the burden of family responsibility and may have more exposure to OPCs, making them a high risk group. In the study on OPPs performed by Singh et al., 76.8% of the female cases and 46.5% of the male cases were from the age group of 16 to 25 years old [19]. Karki et al. similarly reported in their study in eastern Nepal that the majority (65%) of OPP patients were in the 15–30 years age group [20]. Finally the study performed by Srinivas Rao et al. in south Indian hospital revealed that two-thirds of OPP cases were less than 30 years of age [21]. Of all patients, between the age of 14 and 24 years (53.4%) were mostly prone to the incidence which is same as other studies in Nepal (49%) [22] this study only had one case under the age of 14. This poisoning was accidental, this finding was similar with other studies in which most pediatric poisonings are accidental in nature and death is rare [23].

The majority of poisoning cases are women (60%), with a male to female ratio of 1:1.5. These results are consistent with studies performed in several other parts of the world [20, 22, 24]. However, there are additional studies which found that males represented the majority of poisoning patients [25, 26].

Organophosphate poisonings can be absorbed by all routes, including inhalation, ingestion, and dermal absorption [27]. However, the most common route of poisoning in this study was oral ingestion in the suicidal cases (88.9%) followed by accidental poison due to inhalational intake (2.2%), these findings are consistent with other studies [28].

Seasonal variation also alters poisoning statistics. The most number of cases were reported during the spring season (29 cases, 32.2%) followed by rainy (26.6%) and winter season (25.6%). The high incidence during these months is likely attributed to the fact that these months are the most common time to use insecticides and the Ops are easily accessible. This result was dissimilar to another study which showed more cases during summer season [29] and studies reported by Kar et al., 57% of the cases fall in the months between May and August [30].

When the yearly incidence rate is evaluated, there is an increase in OPPs between 2013 (15.3%) and 2014 (42%). This was replicated in a study done by Pokhrel et al. in Nepal where the rate increased from 0.1 to 0.5% in a year [22].

The time from the ingestion of poison to arrival at hospital is very important for patient prognosis and outcome. In this study, there were no deaths reported, and patients presented to the hospital as early as 13 min to as long as 12 h after ingestion.

90% of the patients presented to the hospital within 2 h of ingestion, with a mean time interval of about 1 h 10 min. 80% arrived within 12 h. Most of the patients in this study were from urban, more likely to accesses the health institutions for treatment This observation was also made in other studies [31].

Clinicians used gastric lavage for decontamination of acutely poisoned patients in 30% [27] of patients, which is a rate that was also noted in other studies [24]. Gastric lavage is most effective within 30 min of ingestion, but is thought to be effective up to 4 h post-ingestion, as after this time Ops are no longer seen in the gastrointestinal tract [32, 33]. In this study, at times gastric lavage was used irrespective of the time of ingestion. In 5 patients (19%) gastric lavage was done wrongly and used at inappropriate time increments. Treatment of OP poisoning, secondary to decontamination efforts by gastric lavage, is primarily aimed at reversing the effects of the compound through atropine administration [34]. Atropine is highly effective in antagonizing the actions of OPs at muscarinic receptor sites [35], when the correct diagnosis has been made [36]. In the present study health care providers used decontamination methods in 45.6% of the patients; of these patients, 36.7% received atropine. Out of the 90 patients in this study, 63.3% did not received atropine, which is similar with results seen in other studies (65%) [24]. Even if pralidoxime is effective in reversing the effect of OP, is not available in the study area.

Even though the study is retrospective and might have its own limitations related to documentation problem, the main reasons stated by patients for their suicide attempt were; being family dispute (5.8%), marital disharmony (16.9%), unsuccessful love affair (7.3%), for treatment of a disease (2.4%), being HIV positive (4.8%) and unplanned pregnancy (4.8%). These results were similar to those reported Soysal et al. [37].

## Conclusion

As a developing nation, Ethiopia continues to have OPC poisoning as a significant cause of acute poisonings. The results of this study revealed that women and young adults are more likely to suffer from Ops. In the majority of patients, the ingestion was ingested intentionally as an act of self harm. It is worrisome that the most affected part of the population seems to be the younger generation and that the number of cases is increasing every year. To combat this, it is essential to strengthen the governmental regulations on the availability of OPCs. Additionally, it is essential to strengthen preventive measurable like educating people about the risks of OPCs through health extension workers, establishing and promoting poison control centers, implementing separate toxicological units in hospitals and upgrading peripheral health centers to be able to manage OP poisoning cases. Proper documentations of patient information is essential from health institutions In conclusion, preventing OPPs should be placed as a priority for the Federal Ministry of Health, as it is a preventable cause of emergency department admissions.

## Additional files

**Additional file 1.** Questionnaire.

**Additional file 2.** Utilized data.

## Abbreviations

OP: organophosphorus
OPP: organophosphorus poisoning
OPCs: organophosphorus compounds
OPP/IP: organophosphorus pesticide/insecticides poisoning
OPCP: organophosphorus compound poisoning
