# Supplementary material for: Pattern of acute organophosphorus poisoning at University of Gondar Teaching Hospital, Northwest Ethiopia
Source: BMC Res Notes. 2017 Apr 4;10:149. doi: 10.1186/s13104-017-2464-5 (PMC5381028; doi:10.1186/s13104-017-2464-5)
Supplement: Supplementary file 1 — Additional file 1. Questionnaire. [file 13104_2017_2464_MOESM1_ESM.docx]

Questionnaire for data collection

1. Sex

M 🗖 F🗖

1. Age in years
   1. 11-20 🗖
   2. 21-30🗖
   3. 31-40🗖
   4. 41-50🗖
   5. 51-60🗖
2. Resident
   1. rural 🗖
   2. urban🗖
3. Patient status when coming to the hospital
   1. conscious🗖
   2. unconscious🗖
4. Type of organophosphate poisoning
   1. (specify)------------------------------
5. Dosage form of the poison
   1. solid🗖
   2. liquid🗖
   3. gas 🗖
6. mode of poisoning
   1. Intentional🗖
   2. Unintentional🗖
7. Reasoning of clients taking the poison
   1. Family disharmony🗖
   2. Marital disharmony🗖
   3. Unsuccessful🗖
   4. Domestic violence (pregnant after raped)🗖
   5. For traditional medicine🗖
   6. Mental disorder🗖
   7. Being RVI🗖
   8. Conflict in work area🗖
   9. Financial problem🗖
8. Route of administration
   1. Oral🗖
   2. Inhalation🗖
   3. Contact🗖
   4. others(specify)---------------
9. Time of poison
   1. Day 🗖 ( time in hour--------------------------)
   2. Night🗖 (time in hour------------------------)
10. Time of arrival to the hospital ----------------
11. Source of poisoning
    1. Home🗖
    2. Hotel🗖
    3. work place🗖
    4. others(specify)----------------------
12. Season of ingestion/admission (month)---------------------------------------------
13. Treatment----------------------------------------------------------------------------
14. Duration of hospital stay----------------------------------------------------------
15. Outcome/ cure or death /-----------------------------------------------------------
